# Entomological assessment of hessian fabric transfluthrin vapour emanators for protecting against outdoor-biting *Aedes aegypti* in coastal Tanzania

**DOI:** 10.1371/journal.pone.0299722

**Published:** 2024-05-29

**Authors:** Nicodem J. Govella, Alphonce Assenga, Amos T. Mlwale, Nosrat Mirzai, Eimear Heffernan, Jennie Moriarty, John Wenger, Vincent Corbel, Justin McBeath, Sheila B. Ogoma, Gerry F. Killeen

**Affiliations:** 1 Environmental Health and Ecological Sciences Department, Ifakara Health Institute, Dar es Salaam, United Republic of Tanzania; 2 African Institution of Science and Technology, School of Life Science and Bio-Engineering, The Nelson Mandela, Tengeru, Arusha, United Republic of Tanzania; 3 Institute of Biodiversity, Animal Health and Comparative Medicine, University of Glasgow, Glasgow, United Kingdom; 4 Centre for Research into Atmospheric Chemistry, School of Chemistry, University College Cork, Cork, Republic of Ireland; 5 Environmental Research Institute, University College Cork, Cork, Republic of Ireland; 6 Institut de Recherche pour le Developpement, University of Montpellier, Montpellier, France; 7 Laboratório de Fisiologia e Controle de Artrópodes Vetores (Laficave), Instituto Oswaldo Cruz (IOC), Fundação Oswaldo Cruz (FIOCRUZ), Avenida Brasil, Rio de Janeiro-RJ, Brazil; 8 Envu UK Ltd, Cambridge, Milton, Cambridge, United Kingdom; 9 Abt Associates, Nairobi, Kenya; 10 Department of Vector Biology, Liverpool School of Tropical Medicine, Liverpool, United Kingdom; 11 School of Biological Earth & Environmental Sciences, Environmental Research Institute, University College Cork, Cork, Republic of Ireland; Beni Suef University Faculty of Veterinary Medicine, EGYPT

## Abstract

**Background:**

A low technology emanator device for slowly releasing vapour of the volatile pyrethroid transfluthrin was recently developed in Tanzania that provides robust protection against night biting *Anopheles* and *Culex* vectors of malaria and filariasis for several months. Here these same emanator devices were assessed in Dar es Salaam city, as a means of protection against outdoor-biting *Aedes* (*Stegomia*) *aegypti*, the most important vector of human arboviruses worldwide, in parallel with similar studies in Haiti and Brazil.

**Methods:**

A series of entomological experiments were conducted under field and semi-field conditions, to evaluate whether transfluthrin emanators protect against wild *Ae*. *aegypti*, and also compare the transfluthrin responsiveness of *Ae*. *aegypti* originating from wild-caught eggs to established pyrethroid-susceptible *Ae*. *aegypti* and *Anopheles gambiae* colonies. Preliminary measurements of transfluthrin vapour concentration in air samples collected near treated emanators were conducted by gas chromatography-mass spectrometry.

**Results:**

Two full field experiments with four different emanator designs and three different transfluthrin formulations consistently indicated negligible reduction of human landing rates by wild *Ae*. *aegypti*. Under semi-field conditions in large cages, 50 to 60% reductions of landing rates were observed, regardless of which transfluthrin dose, capture method, emanator placement position, or source of mosquitoes (mildly pyrethroid resistant wild caught *Ae*. *aegypti* or pyrethroid-susceptible colonies of *Ae*. *aegypti* and *An*. *gambiae*) was used. Air samples collected immediately downwind from an emanator treated with the highest transfluthrin dose (15g), contained 12 to 19 μg/m^3^ transfluthrin vapour.

**Conclusions:**

It appears unlikely that the moderate levels of pyrethroid resistance observed in wild *Ae*. *aegypti* can explain the modest-to-undetectable levels of protection exhibited. While potential inhalation exposure could be of concern for the highest (15g) dose evaluated, 3g of transfluthrin appears sufficient to achieve the modest levels of protection that were demonstrated entomologically. While the generally low levels of protection against *Aedes* reported here from Tanzania, and from similar entomological studies in Haiti and Brazil, are discouraging, complementary social science studies in Haiti and Brazil suggest end-users perceive valuable levels of protection against mosquitoes. It therefore remains unclear whether transfluthrin emanators have potential for protecting against *Aedes* vectors of important human arboviruses.

## Background

Despite having abundant populations of *Aedes aegypti* and suitable conditions for transmission of Dengue, Chikungunya and Zika viruses [1,2], these diseases received relatively little attention in Tanzania until 2014 when the first large dengue outbreak occurred in urban Dar es Salaam [3,4]. This coincided with Zika epidemics in South and Central America [5], which then became a subject of public health interest to both policy-makers and researchers [1,4,6–9]. Local *Aedes aegypti* populations in Dar es Salaam typically bite people outdoors during daytime hours (Personal observations), so there are limits to the degree of protection that may be provided by measures designed to protect people while indoors, such as insecticide-treated bed nets, spraying insecticides inside houses or mosquito-proofed housing [2].

Nevertheless, outdoor exposure to mosquito bites may be reduced through the dispersal of volatile compounds with insecticidal properties, generically referred to as *spatial repellents*, into the air around otherwise vulnerable humans [10,11]. The term *repellent*, however, is often something of a misnomer because some of the most important examples, notably volatile pyrethroids like transfluthin, exhibit more complex modes of action than merely deterring mosquitoes from biting, and can confuse, incapacitate or kill mosquitoes at higher concentrations [12–16]. Regardless of their diverse modes of action, however, most existing products only protect against mosquitoes for short durations, most of them lasting for only a few hours per application or dispensing dose [17]. Correspondingly, sustaining continuous protection through repeated reapplication and replacement is probably impractical and unaffordable for most people living in low-income countries like Tanzania [17–19], while some combustion-based coil emanator formulations for releasing volatile insecticides into the air may even be hazardous [20,21].

Recently, however, a low technology emanator, which passively releases vapour of the spatial repellent transfluthrin far more slowly under ambient temperature conditions without any electricity or other power source, has been developed and field tested in Tanzania as a means of protection against night biting *Anopheles* and *Culex* spp. vectors of malaria and filariasis. In Dar es Salaam, on the coast of Tanzania, it provided >90% protection against both genera for at least 4 months after treatment [22], while in a rural inland setting it provided >75% protection for 6 months and no diversionary effect to nearby non-users was detected [23]. However, these transfluthrin emanator devices have not been evaluated against the day-time biting *Aedes* that transmit Dengue, Chikungunya, Yellow fever and Zika viruses. Furthermore, potential exposure of human users to transfluthrin vapour have yet to be assessed for this type of passive emanator.

This study therefore set out to address these knowledge gaps in coastal Tanzania by assessing whether this new transfluthrin emanator could provide protection against wild *Aedes* populations without diverting them to attack unprotected non-users nearby. It also aimed to assess whether a commercializable, pre-formulated, emulsifiable concentrate provided equivalent levels of efficacy to the treatment emulsion prepared from technical grade transfluthrin and the locally available generic detergent products that had been used in previous studies. Also, some limited measurements were made of the concentration of transfluthrin vapour in the air space around emanators treated with the maximum dose under representative semi-field conditions, to get a preliminary idea of what range of exposure rates end users might experience.

## Methods

### Study areas and experimental sites

This article reports all the results obtained from the Tanzanian site of a two-country study carried out in the cities of Dar es Salaam in the United Republic of Tanzania and Port-au-Prince in the Republic of Haiti, the interpretation of which is also informed by a separate but nevertheless similar study in Brazil that the authors in question have freely shared the results of (Alvaro Eiras, personal communication). All procedures for this study were harmonized with those for a similar entomological assessment of transfluthrin emanator efficacy [24] and a complementary social science assessments of end-user perceptions [25], which were carried out in parallel in urban Haiti.

**Fig 1:**
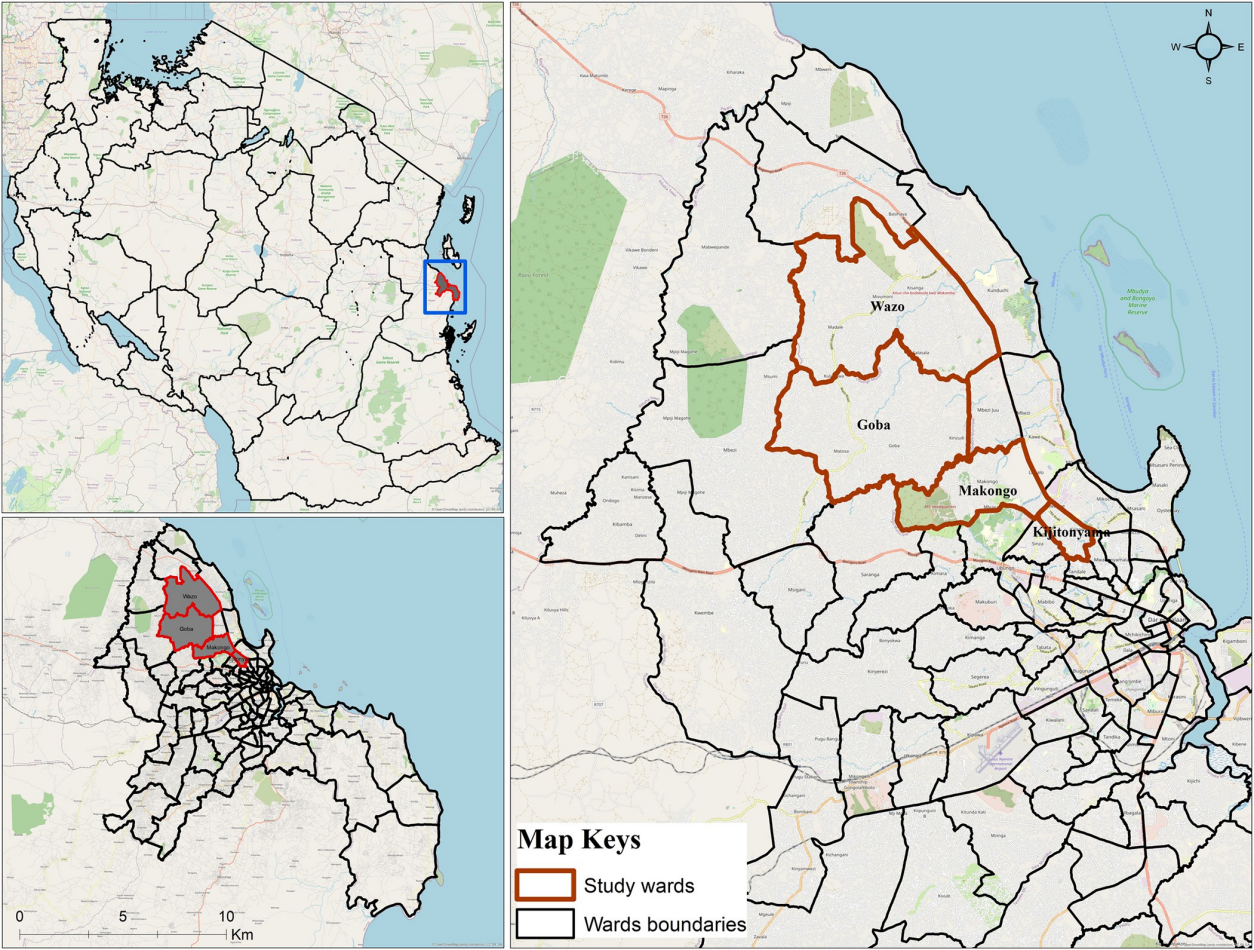
Study area and experimental sites in Dar es Salaam. The green polygons represent five wards within the Kinondoni Municipality within which eight sampling blocks were located. This map was produced with ArcGIS ©2023 Esri, licensed to the Ifakara Health Institute, using a base map obtained from OpenStreetMap © OpenStreetMap contributors, under the Open Database License.

This study was conducted in Dar es Salaam (Fig 1), the largest city and commercial hub in Tanzania. Dar es Salaam has an area of 1,339km^2^ with a population of 4.4 million people according to the last census [26]. It is located along the shore of Indian Ocean at 55 m above sea level. Administratively, it consists of five districts: Kinondoni in the north, Ilala in the centre, Ubungo, Temeke in the south and Kigamboni in the east. The city is characterised by high concentrations of trade, manufacturing, coupled with unplanned settlement, poor drainage and sanitation system. Climatically, two rainy seasons are exhibited: short rains (October to December) and long rains (March to May) with 25.9°C average temperature. Detailed description of the area can be found elsewhere [3,27]. Along with vectors of malaria (*Anopheles gambiae s*.*l*, *Anopheles funestus*) and filariasis (*Culex spp*.), *Aedes* mosquitoes are also widespread throughout Dar es Salaam [3,6,28]. Open water storage containers, uncollected solid waste disposal (discarded plastic and used tyres) and flowerpots are the most productive breeding habitats of *Aedes* mosquitoes in Dar es Salaam [6,29]. Apart from being widespread, these day-active mosquitoes exhibit reduced susceptibility to pyrethroid insecticides [6], potentially undermining efforts to control them with this crucially important class of public health insecticides [30–32]. Experimental sites for this study were identified within the Kinondoni district because a previous study confirmed this district had reasonably high numbers of *Ae*. *aegypti* [3]. A total of eight sites distributed within five wards of Kinondoni (Mikocheni, Kijitonyama, Goba, Wazo and Makongo) were purposively selected (Fig 1). Criteria used for the selection of sites included: 1) open field ground measuring at least 50 × 100m and situated in between human settlements, 2) Having sufficient catches of *Aedes* mosquitoes to allow statistical detection of the effect of the treatment.

### Formulation of transfluthrin treated strips

**Fig 2:**
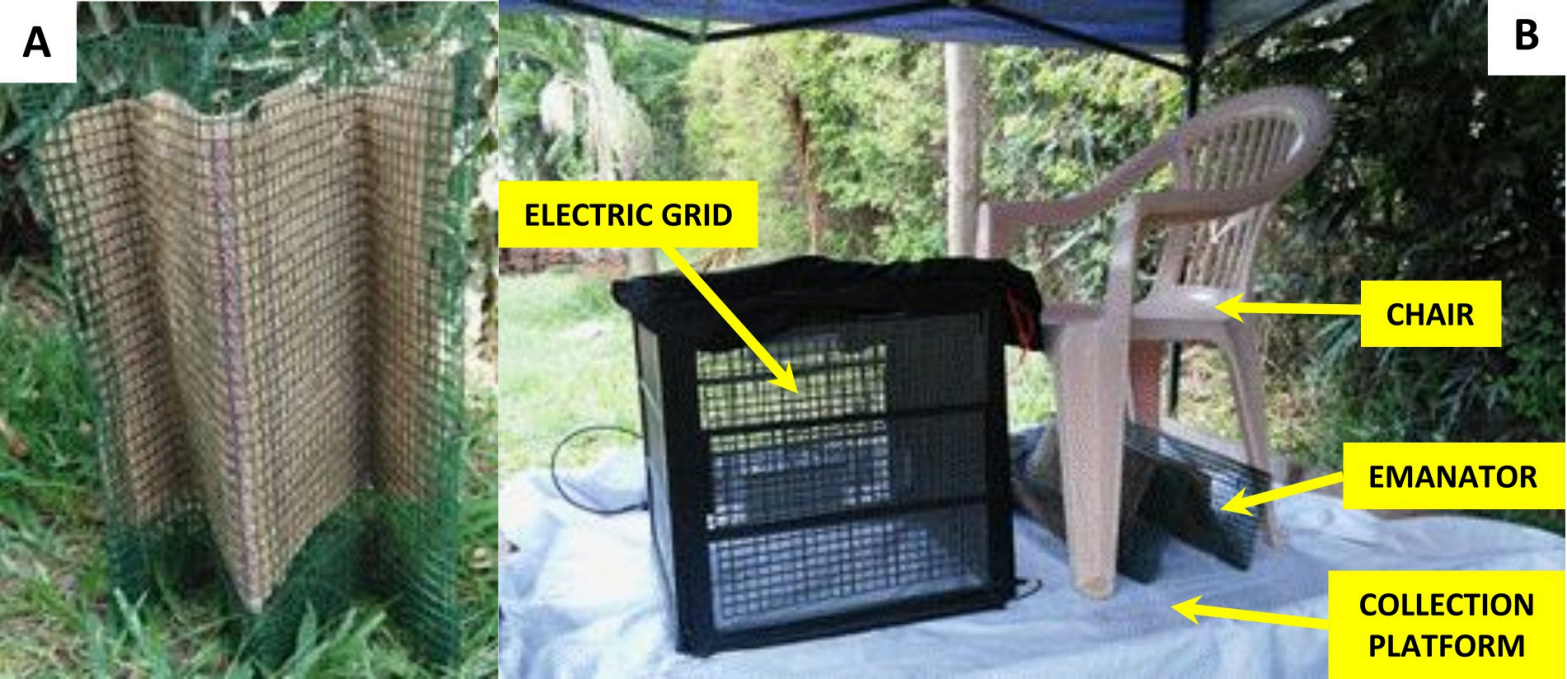
Design and evaluation of the M-shaped emanator. **A**: Hessian strip enclosed within a PVC wire-mesh to provide prevent dermal contact with the treated strip and allow folding into semi-rigid zig-zag shape. **B:** Illustrates how the emanator was placed underneath the chair of a seated participant with his/her feet placed inside a mosquito electrocuting trap [35,36].

Strips of hessian fabric each measuring 70 × 40 cm from jute bags were bought from the local market. Strips were treated with 99% technical grade transfluthrin (Bayer AG, Environmental Sciences at the time, now trading as Envu AG, Germany). An emulsified mixture containing 3g of transfluthrin, 90ml of Axion liquid detergent (Orbit Chemical Industries Ltd, Nairobi and Colgate-Palmolive East Africa Ltd) and 400ml of water was prepared and then soaked into each strip as previously described [23]. Each strip was then left to dry indoors at ambient temperature. Control strips were also soaked into equal mixture of water and detergent, but without transfluthrin. Dried strips were each wrapped within a PVC coated wire-mesh (Goodonehouse, Monkey Wire Mesh, Africano, Limited, China) to form an M-shape emanator (Fig 2A). The PVC-coated wire-mesh cover was designed to prevent dermal contact of participants and researchers with the treated strips.

### Measuring mosquito landing rates

Unlike previous protective efficacy assessments of such transfluthrin emanators using traditional human landing catches (HLC) [22,23,33,34], which inevitably expose those volunteers to potentially infectious bites from wild mosquitoes under full field conditions [32], here human mosquito landing rates were measured with recently developed, field validated user-insulated mosquito electrocuting traps (METs) placed around the feet of volunteers seated in chairs (Fig 2B), who were fully protected against mosquito bites with protective clothing. The details of how these electric grid devices may be used to sample host-seeking mosquitoes are described elsewhere [35–39].

### Longitudinal assessment of transfluthrin emanators protection against wild *Aedes aegypti*

Eight different open field sites (each measuring approximately 120 × 70m) were identified within urban Dar es Salaam and used as study areas over the course of this experiment. Each site had its own pair of treated and untreated hessian strips. Three consecutive days of day-time mosquito biting collection were conducted weekly per site. This means that a total of eight weeks (24 days of sampling) were needed to make a complete round of experimental replication through all eight sites. A total of four rounds of experimental replications were completed over the course of this experiment, with data collection running from 9^th^ May 2017 to 11^th^ January 2018. This timing allowed mosquito sampling across rainy and dry seasons. During the experiment, one user assigned a treated strip and another assigned and untreated one were spaced 80m apart from each other within the open field to limit possible crossover effects arising from transfluthrin vapour passing from one station to the other. These strips were placed under a chair of the users, just behind users’ feet, which were enclosed within the MET structure (Fig 2B).

Throughout the experiment, the strips, users and MET devices were placed on a raised platform with a white plastic covering to make mosquitoes easy to see and recover (Fig 2B), underneath a shelter with a plastic sheeting roof supported by four metal poles, so that they were adequately protected against direct sunlight and rainfall. At night, and in between periods of active experimentation, each strip was placed inside a card box and was stored indoors at room temperature, with treated and untreated strips stored in separate rooms.

In order to detect any effect of users of treated transfluthrin emanators upon nearby non-users of the emanators, two METs occupied by non-users were both placed at one of six pre-defined distances (2m, 5m, 10m, 15m, 20m, and 25m) and angles (0°, 60°, 120°, 180°, 240° and 300°) from each of the two matched METs occupied by emanators users (Fig 2B). These distance-angle combinations were each randomly selected without replacement to each of the six hour-long sampling intervals in each day of experiment, similarly to previous experiments with nocturnal mosquitoes in rural Tanzania [23] but designed to match the quite different activity peaks anticipated for *Ae*. *aegypti*, and therefore split across one three-hour period in the morning (06:00–07:00, 07:00–08:00, 08:00–09:00) and another in the evening (16:00–17:00, 17:00–18:00, 18:00–19:00). Initially, the evening shift collection was started at 15:00–16:00, but this was amended after the first six weeks of collection because of high temperatures and low mosquito catches.

Collection was conducted for 45 minutes of each hour period, with 15 minutes allowed for rest, refreshment, and collecting mosquitoes from the grids. At the end of each hour of collection, each pair of volunteers at each location of the open field site (one strip user and his/her allocated non-user counterpart nearby) exchanged positions, so that each volunteer spent an equal amount of time at the user and non-user positions, thus minimizing potential biases associated with the differential attractiveness of individuals to mosquitoes.

Three different strip treatment arrangements were allocated at random, without replacement, to each day of a three-day weekly replication cycle. These are described as T+C, C+T and C+C, where T and C respectively stand for Treatment and Control and the first letter describing the type of emanator allocated to the southern location within the open field site whereas the second letter was allocated to the northern one. For example, if the T+C arrangement was allocated to the first working day of the week, this meant that the southernmost location of the two spaced 80m apart within the open field site was occupied with a volunteer using a strip treated with transfluthrin, while the other to the north was provided a negative control strip lacking any transfluthrin. The C+T arrangement denoted the exact opposite orientation to the allocation of treated and untreated strips, while C+C denoted the allocation of placebo-treated negative control strips to both users.

During the experiment, all mosquitoes captured by electrocuting trap by each volunteer and in each hour were placed in a separately labelled paper cup. After morning or evening shift of experiments, mosquitoes in each paper cup were first killed with ethanol, sorted morphologically by genus and abdominal status and then counted. All these observations were then entered into a pre-designed, paper-based data collection form, and then entered, cleaned and linked using a standardized entomological data informatic system as previously described [40].

Data was analysed by fitting generalised linear mixed models (GLMMs) using R statistical software version 3.2.1, with the *lme4* package augmented within *matrix*. Because the protective efficacy of transfluthrin repellent may be sensitive to temperature [23], analysis was restricted only to data that were collected at temperatures > 22°C, encompassing a total of 455 captured female *Aedes aegypti*. Determining the effect of treatment upon emanator users and nearby non-users of the treated emanators, users of treated strips in the arrangement of TC at 0m distance and associated nearby non-users at distinct distances (2m, 5m, 10m, 15m, 20m and 25m) was treated as fixed categorical factor in a GLMM with users of untreated control (C) strips from both the C+C and C+T arrangements, as well as their associated nearby non-users all pooled into a single reference group. The catches of female *Aedes aegypti* from each hour of collection at each MET were fitted as the response variable, assuming a Poisson distribution. Note that although models assuming a negative binomial distribution for the dependent mosquito catch variable were also assessed, these consistently yield poorer goodness of fit statistics and often failed to converge. Angle by distance combination nested within location of collection nested within field site was treated as one multi-level random effect in the model, while date was treated as another independent simple random effect. In case of assessing the effect of time on protection efficacy of the treated emanators, a continuous numerical interaction term, representing the number of days since treatment of the strips conditional upon the strips being treated with transfluthrin, was treated as a continuous covariate with location nested within field site and angle by distance combination as a multi-level random effect, while date was treated as another independent simple random effect.

### Comparisons of alternative emanator designs and transfluthrin formulations against wild *Aedes aegypti*

**Fig 3:**
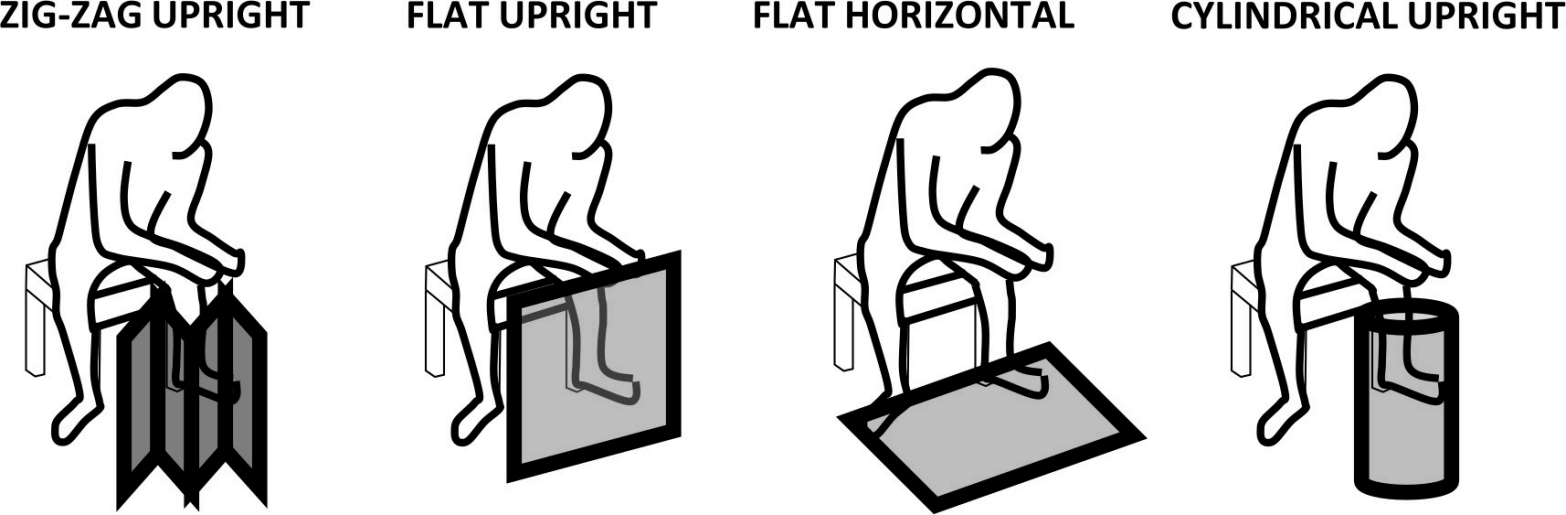
A schematic illustration of the four different emanator prototype designs evaluated against wild *Aedes aegypti* populations in Tageta, Dar es Salaam. (1) The established rectangular design, folded into a self-standing zig-zag shape placed immediately in front of the user, (2) the same prototype left flat and unfolded and stood up vertically by leaning up against the user’s chair or other nearby object, (3) the same prototype laid out flat in a horizontal orientation, and (4) the same prototype wrapped around itself into a cylindrical shape that is also self-supporting in a vertical orientation.

Subsequent troubleshooting experiments were undertaken to assess whether alternative emanator designs and treatment formulations might improve the level of protective efficacy observed and help shed light on possible causes of the apparently limited protection provided by treated emanators over much of the course of the initial longitudinal evaluation described above. To evaluate and compare the efficacy of four different emanator prototype formats (Fig 3) and three different treatment formulations, a replicated Latin square design was used. One complete replicate of the following experimental design was completed, with all 16 combinations of emanator design and treatment formulation rotated through in a single block of 16 catching stations in Tageta, Dar es Salaam over 16 days (Fig 1). The four emanator designs evaluated (Fig 3) and four replicate emanators of each of these four designs were each treated with one of the four following formulations, allocated at random: (1) a placebo emulsion containing only water and detergent, (2) the same diluent used to emulsify 3g of transfluthrin active ingredient from a technical concentrate (TC) formulation received from Bayer AG (Now trading as Envu AG) the previous year, (3) a similar emulsion prepared with a new batch of transfluthrin TC formulation provided by Bayer/Envu immediately before the study, and (4) a new liquid emulsion based on dilution of the same amount of active ingredient from a formulated (20%) Emulsifiable Concentrate (EC) provided by Bayer/Envu.

Sixteen human volunteers were assigned to one of 16 catching stations and mosquitoes were collected using the same MET [35–39] methodology described above (Fig 2) over 16 days. On the first day, each of the 16 emanator format by strip treatment combinations described above were assigned at random to one of these numbered catching stations and then rotated in sequence through all 16 catching stations over 16 days. This rotation procedure was repeated once, representing two full replicates of this Latin square design distributed over 32 days of experimentation between the 14^th^ of May and the 24^th^ of June 2018.

Because no effect upon nearby non-users was observed in the preceding experiments described above, further investigation into this possibility were not considered important and such large open field sites were no longer considered necessary. Catching stations were therefore placed in among houses to maximize the densities of *Aedes aegypti*, which thrive in peri-domestic living areas but don’t fly far from them. The exact locations chosen for these catching stations were also chosen to minimize disturbance to the residents and risks of accidental contact with the METs.

Note that each volunteer was allocated to a single, fixed station at each block for the full duration of the experiment, so that these two underlying causes of variation in capture rates associated with station and volunteer are combined into a single source of variance that could be captured with a single random effect and maximum statistical power in the analysis. The effect sizes were estimated as relative rates at which *Ae*. *aeygpti* landed on volunteers using treated emanators, as measured with METs [35–39], compared to the volunteers using placebo-treated emanators, using essentially the same GLMM statistical methodology described above.

### Comparisons of alternative mosquito capture methods and emanator deployment positions against *Aedes aegypti* colony mosquitoes

Two chambers of the semi-field system (each measuring 9 × 29m) which were separated by a middle chamber measuring 3 × 9m were used. Of the two chambers, one was assigned an emanator strip treated with 3g of transfluthrin while the other was assigned a placebo-treated negative control strip. The M-shaped emanator was placed underneath chair just behind the feet of a seated volunteer conducting either the MET (Fig 2B) or HLC method for collecting mosquitoes. On the first day, one of these two sampling methods was randomly selected and then assigned to both treatment and control chambers for the whole day, following which the capture method was alternated every second day, with treatments swapped over every day. This way, it was possible to compare the estimate of protective efficacy from the two methods over a period of 12 days of experimentation, carried out between the 6^th^ and 23^rd^ of September 2018. Each day, a total of 400 adult female *Ae*. *aegypti* mosquitoes from the fully pyrethroid-susceptible Bagomoyo strain laboratory colony were released into each chamber.

The effect of placing the emanator in front of the user’s legs, rather than under the user’s chair, upon estimated protective efficacy was assessed through an otherwise identical study design to the above (12 days of experimentation carried out between the 4^th^ and 24^th^ of November 2018), but with deployment position as the experimentally controlled factor of interest that was assigned at random on the first day but then exchanged between test chambers on each subsequent day.

The effects of capture method and emanator position upon relative landing rates of *Ae*. *aeygpti* were estimated by fitting two separate GLMMs to the separate data sets obtained through the two distinct experiments described above, exactly as described in the preceding subsection except that categorical independent variable of interest was either the capture method used (MET versus HLC) or emanator position relative to the user (In front of the user’s legs versus under the user’s chair).

### Comparison of susceptibility to conventional solid phase pyrethroid and responsiveness to transfluthrin vapour at a range of doses in wild mosquito populations and laboratory colonies

The generally unsatisfactory levels of protective efficacy exhibited by transfluthrin treated emanators in the experiments described above motivated experimental assessment of the two following explanatory hypotheses: (1) wild field populations of *Ae*. *aegypti* may be resistant to pyrethroids and consequently less responsive to transfluthrin vapour [32] than fully-susceptible colony mosquitoes, and (2) higher doses of active ingredient may be required against *Ae*. *aegypti* than against the *Anopheles* and *Culex* mosquitoes that these devices were originally optimized for [23].

In order to assess whether pre-existing pyrethroid resistance could diminish responsiveness to transfluthrin treated emanators [30–32], further experiments were conducted to compare their protective efficacy across a wide range of doses when tested against three different mosquito populations with different levels of susceptibility to pyrethroids: (1) multiple independent collections (at least one distinct one per experimental replicate, to preclude pseudo-replication) from mildly resistant local field populations of *Ae*. *aegypti* in Dar es Salaam, (2) *Ae*. *aegypti* sourced from a fully pyrethroid-susceptible laboratory colony of the Bagomoyo strain that originated from coastal Tanzania, and (3) *Anopheles gambiae* sourced from a fully pyrethroid-susceptible laboratory colony of the Kisumu strain that originated from western Kenya.

Standard World Health Organization (WHO) assays [41] were used to assess the susceptibility of *Aedes aegypti* mosquitoes to conventional solid phase pyrethroids using reference treated papers supplied by the WHO reference laboratory (kindly provided by Dr David Weetman at the Liverpool School of Tropical Medicine).

To enable direct comparison, F2 generation *Ae*. *aegypti* originating from wild-caught eggs from the Dar es Salaam study site described above were reared in captivity in a large-cage semi-field facility, and then tested for susceptibility to permethrin and deltamethrin in parallel with fully pyrethroid-susceptible stock from established colonies of *Ae*. *aegypti* and *An*. *gambiae* that were reared alongside them under the same conditions. For the field-sourced mosquitoes, each experimental replicate included at least one distinct independent collection, which was not used in any other replicate, to preclude pseudo-replication arising from genetic relatedness within collected egg batches.

The bioassays were conducted with papers treated with 0.75% permethrin and 0.05% deltamethrin, with oil impregnated papers as controls. For each test, four replicates of 25 female *Ae*. *aegypti* between 3 and 5 days old were exposed to the treated papers for 1 hr and mortality was recorded after 24 hrs of exposure as per WHO guidelines [41]. Mean mortality was estimated using GLMMs lacking an intercept fitted with the *lme4* package in R, with the proportion that had died treated as the dependent variable with a binomial distribution and logit link function, while insecticide treatment was included as a fixed categorical independent variable and replicate was included as a random effect. The results were represented graphically using dot plots of mean mortality rates for individual replicates overlain with box plots representing estimated medians plus first and third quartiles.

To evaluate the protective efficacy of emanators treated with various doses of transfluthrin, the same MET method (Fig 2) was used inside a large-cage semi-field facility with insectary-reared mosquitoes from the three different wild and colony sources described above. Five doses (3g, 6g, 9g, 12g and 15g) of transfluthrin TC formulation were tested, and each test was replicated four times. Two treatment arrangements were used: T+C and C+C. One of the two arrangements was randomly selected without replacement for the first day of the two-day experimental cycle required to complete a single replicate for each dose, with the remaining arrangement applied on the second day. For example, if the T+C arrangement was randomly selected for the first day, this means that one of the two locations within the semi-field large cage system (13m apart at the northern and southern ends), was occupied by a volunteer using a strip treated with transfluthrin, while the other location was occupied by a volunteer with a placebo-treated negative control strip. The C+C arrangement was selected for the second day of the replicate where volunteers in both the northern and southern locations used negative control strips lacking any active ingredient. Alternatively, if the C+C arrangement was selected first, then the T+C arrangement followed on the second day. Note that for the T+C arrangement, the assignment of the treatment and control emanators to the northern and southern locations were randomly assigned afresh on each experimental day. However, volunteers remained at a given location throughout the experiment once they had been assigned to one location or the other at the outset, so that these two potential sources of variation could be parsimoniously accounted for as a single source of variance in the analysis. The order in which each dose was assessed within each round of replication was also randomly replicated without replacement.

Effect sizes were estimated as relative rates at which *Ae*. *aeygpti* landed on volunteers using treated emanators compared to volunteers using untreated placebo emanator devices. All estimated mean relative landing rates and their 95% confidence intervals were obtained by fitting a separate GLMM to the data from each of the three different mosquito sources, with the number of mosquitoes caught representing the dependent variable with a Poisson distribution and the various treatment doses included as the categorical independent variable of interest with the placebo-treated negative control strips as the reference group, while date, location and replicate were included as random effects.

### Measurement of transfluthrin vapour concentration in the air around treated emanators

Samples were collected using inert coated stainless-steel tubes packed with 200 mg Tenax TA adsorbent (Markes International, UK). The tubes were pre-conditioned and sealed with brass long-term storage caps, which have been validated for storage of sampled/blank tubes for up to 27 months. Tubes were opened at the sampling location and connected to a low-flow sampling pump (Markes Acti-VOC) set to operate at a flow rate of 200 cm^3^/min. The pump was calibrated before and after sampling using a flow meter (7000 GC Flowmeter, Ellutia Chromatography Solutions, UK). After sampling for the desired length of time, the tube was disconnected and sealed with the brass caps. Tubes were stored at room temperature until analysis.

**Fig 4:**
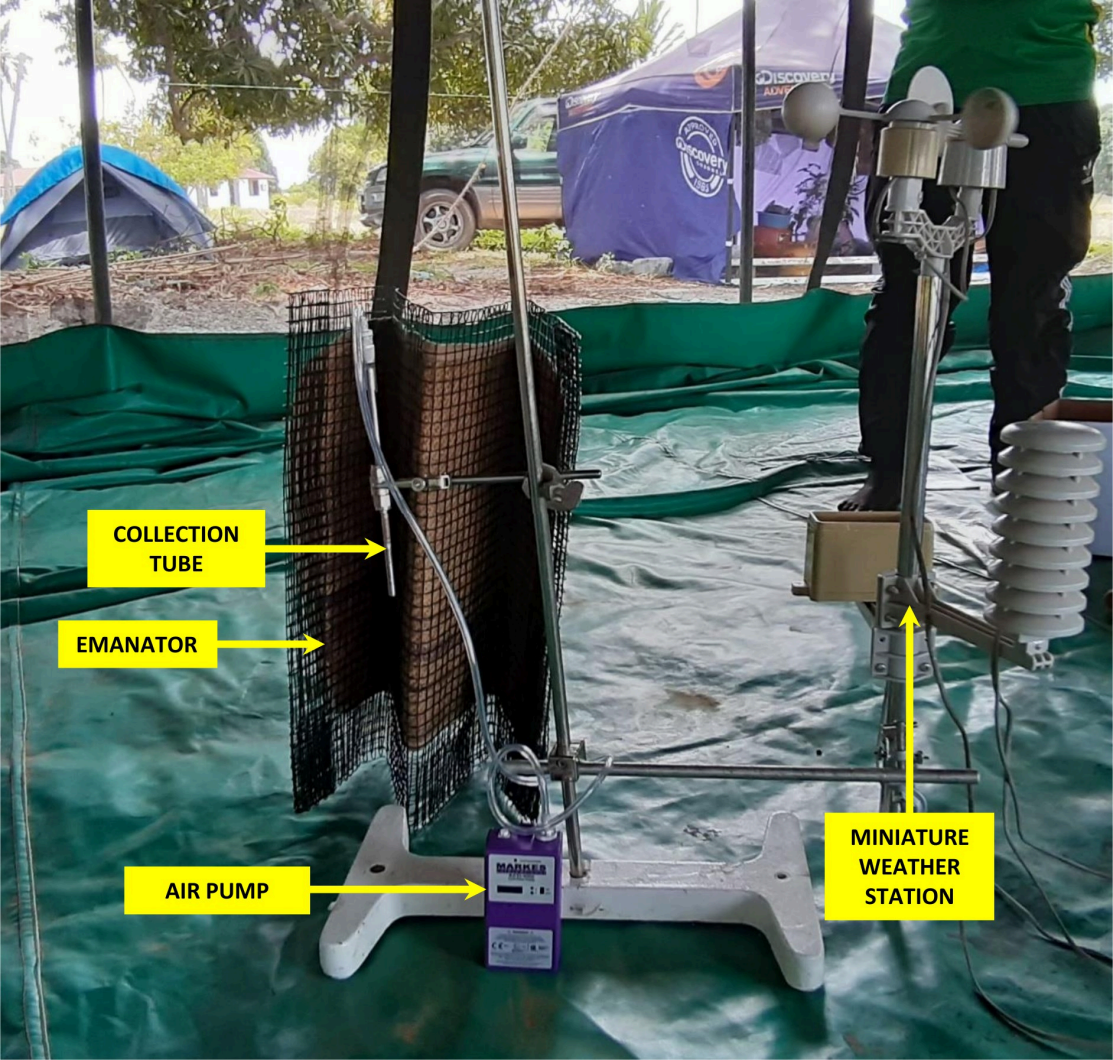
Experimental set-up for measuring transfluthrin vapour near a treated emanator strip inside a semi-field large-cage system.

Sampling of emanated transfluthrin vapour was performed at the IHI Bagamoyo Branch facilities, just north of Dar es Salaam in a large cage semi-field system, containing a hessian fabric emanator sandwiched in a foldable wire mesh. The emanator was treated with 15g of transfluthrin and the sample collection tube was placed about 10 cm downwind from the emanator (Fig 4), to represent the highest possible human exposure to airborne transfluthrin for someone sleeping with their nose and mouth right beside the emanator, as observed for one infant in an end-user household in Haiti [25]. In some experiments, two sample tubes were used in series to test the trapping efficiency of the tube. Temperature was measured with a miniature weather station.

Four different transfluthrin vapour experiments were performed with the semi-field system in Bagamoyo, as detailed in Table 1 in the results section. The two-tube system was used in the two experiments with longer duration. Three field control samples were also prepared by injecting small amounts of a transfluthrin (in acetone solution) onto the gauze at the sampling end of the tube while pumping at 200 cm^3^/min for approximately 5 minutes.

**Table 1:** Details of the transfluthrin vapour sampling experiments conducted inside a large cage semi-field system as illustrated in Fig 4 and the analytical results obtained by gas chromatography with mass spectroscopy detection.

Date	Experiment	Sampling period	Temperature range (°C)	Volume of air sampled (m^3^)	Transfluthrin	
					(ng)	(ug/m^3^)
20/2/2019	Sampling duration of approximately 6 hours, with a second collection tube in tandem to capture any breakthrough from the first.	12:14–18:09	32–30	0.0710	1374	19.35
21/2/2019	Sampling duration of approximately 1 hour	10:22–11:29	28–29	0.0134	170	12.65
21/2/2019	Sampling duration of approximately 1 hour	11:42–12:53	29–30	0.0142	221	15.57
21/2/2019	Sampling duration of approximately 3 hours, with a second collection tube in tandem to capture any breakthrough from the first.	14:11–17:05	28–30	0.0348	630	18.11

Quantitative analysis of transfluthrin was conducted using a Unity 2 thermal desorber (Markes International, UK) connected by a heated transfer line to a GC-MS (Agilent 5977B). The transfer line was connected directly to the analytical column through the heated injector body with the liner removed. The temperatures and flows used in the analytical method were very similar to those reported by Martin *et al* [42].

Tubes were dry purged for 2 min using a 1/20 split. A two-stage desorption was employed, the first stage desorption was performed at 150°C and held for 5 min, the second stage desorption was performed at 300°C and held for 5 min. Trap flow was held at 50 ml/min with a split flow of 10 ml/min during tube desorption. The trap used was a materials emissions trap and this was held at 30°C during tube desorption. Prior to trap desorption a 2 min pre-trap fire purge was performed with a 1/50 split. Trap desorption was performed at 30–300°C at a rate of 24°C/min and held for 5 min in splitless mode.

An Agilent Technologies 7890A GC coupled with 5977B series MS was used to separate volatile compounds connected to a 5977B MSD. The GC-MS was fitted with a HP-5MS ultra inert fused silica column (Agilent, Germany; 30 m × 0.25 mm × 0.25 μm film thickness) and helium (UHP) used as the carrier gas, with a flow rate of 1.2ml/min.

The oven programme temperature was 40°C, held for 5 min, increased to 320°C at 20°C/min and held at 320°C, with a total run time 24 min. Injector temperature was set at 250°C. The ion source temperature was 220°C and the interface temperature was set at 280°C. An auto-tune of the GC-MS was carried out prior to the analysis to ensure optimal performance. A set of external standards was run at the start and end of the sample set and abundances were compared to known amounts to ensure that both separation and MS detection was performing within specification. Electron ionisation (70 eV) was used and the MS was operated in both full scan (70–360 amu) and selected ion monitoring (SIM) modes. Quantitation of transfluthrin was performed using the ion at *m/z* 163, while ions at *m/z* 165, 127 and 91 were used for qualification purposes.

All samples and field controls were analysed, along with the unused tubes that travelled to Tanzania, which effectively served as “travel blanks”. Laboratory calibration curves were generated by quantitatively loading diluted stock solution, ranging in concentration from 1.0 to 900 ng of transfluthrin per μL of acetone. The amount of transfluthrin on each tube was determined using the response at *m/z* 163 and the calibration curves. In line with best practice, the samples, controls and travel blanks were analysed in random order. The negligible amounts of transfluthrin detected in the second, or “breakthrough”, tubes confirmed that the adsorbent was highly efficient at trapping transfluthrin.

### Ethical considerations

The procedures for this study were reviewed and approved by the Institutional Review Board of the Ifakara Health Institute (Refs. IHI/IRB/No. 016–2016 and IHI/IRB/AMM/No. 12–2017) and the National Research Ethics Committee of the National Medical Research Institute (Refs. NIMR/HQ/R.8c/Vol. I/567 and Vol. II/906) in the United Republic of Tanzania, as well as the Research Ethics Committee of the Liverpool School of Tropical Medicine (Ref. 16–037). Permission to publish this study was kindly provided by Director General of the National Institute for Medical Research of the United Republic of Tanzania.

At the outset of the study, the concentrations of tranfluthrin vapour released by these emanator devices had previously been measured as only 0.00013 mg/m^3^ [23], which compares very well (<1/1000th) with its registered acceptable exposure concentration of 0.5 mg/m^3^ for the European Union [43]. Inhalation exposure to transfluthrin was therefore considered to present negligible risk to participants at the outset of this study.

The MET device is designed to kill mosquitoes before they can bite, so human volunteers sitting within it are not exposed to increased risk of mosquito-borne infections [35–39]. Each participant in mosquito landing catches sat on a chair with his or her legs protected within the square plastic frame of the MET, while the rest of body was protected from mosquito bites by a wearing hat with a netting curtain, a long sleeve shirt and gloves (Right hand panel of Fig 2). From within the square PVC/wooden frame is lined up with insulating plastic fiber mesh which serves not only for protection of mosquito entry, but also prevent volunteer’s limbs from making contact with the exterior electrified wires of the MET device [35–39]. Furthermore, only adult males (≥18 years) and adult females of non-child-bearing age (≥50 years) were recruited as participants in mosquito landing catches, to comprehensively avoid any risk of infection with Zika, malaria or any other vector borne pathogen to which pregnant women are particularly vulnerable.

All participants recruited into this study, between March 2017 and September 2018, were fully informed of these potential risks and benefits of participation in the study, as well as their freedom to withdraw at any stage, and were given every opportunity to ask any questions they had before informed consent was documented in writing. Although several of the investigator knew the participants by name and could therefore identify them as individuals in the datasets based on their recorded initials, no person outside the research team could do so. Correspondingly, no personally identifiable data or images are presented in this publication.

## Results

### Longitudinal assessment of transfluthrin emanators protection against wild *Aedes aegypti*

**Fig 5:**
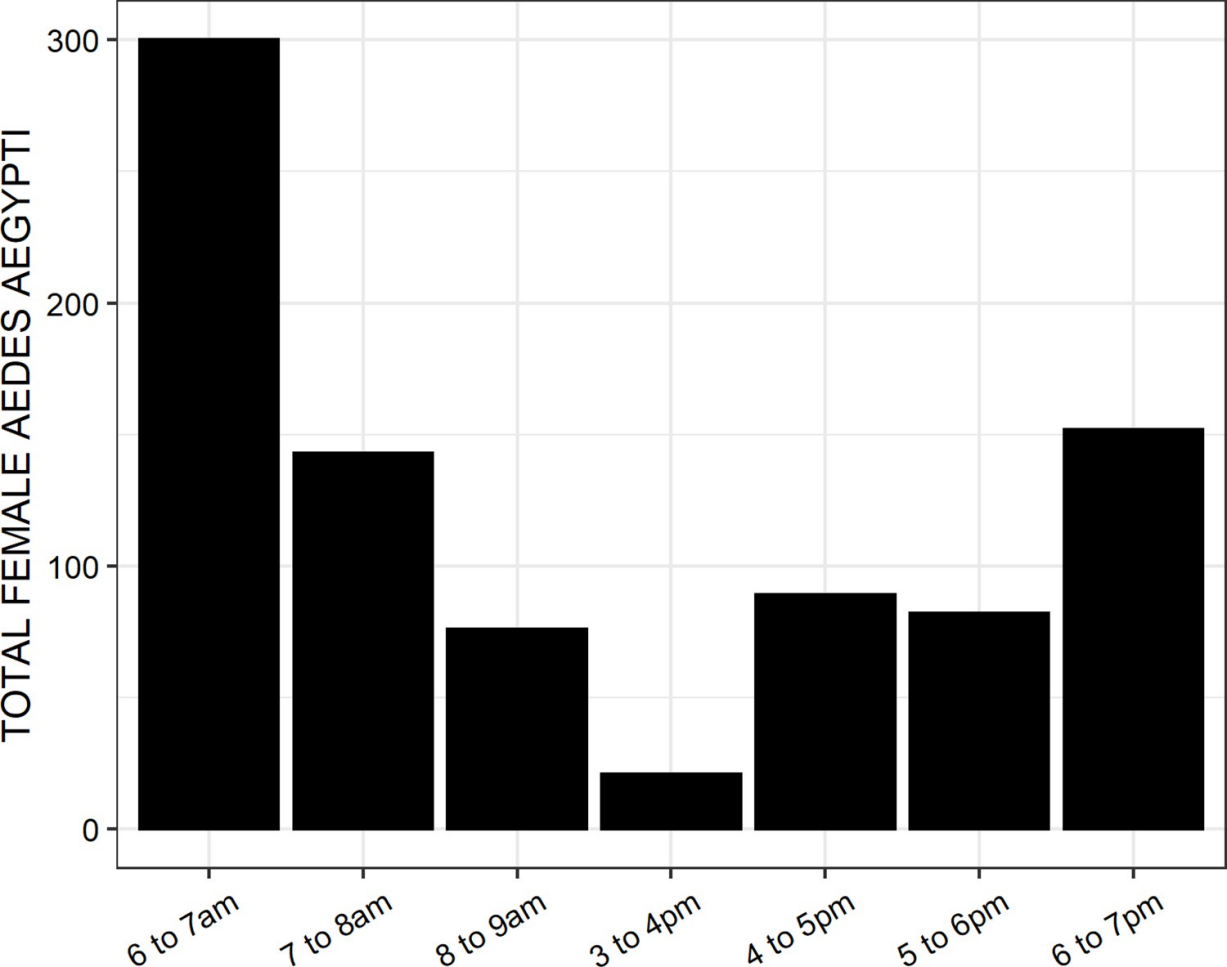
Distribution of human landing activity by wild *Aedes aegypti* across different times of the mornings and evenings in urban Dar es Salaam, Tanzania.

A total of 879 female *Aedes aegypti* was collected over the course 96 days of experimentation distributed across 8 months. This represents an average of 2.3 mosquitoes per participant per day of experimentation, so a quite low landing rate overall. Of the total 863 (98.2%) and 16 (1.8%) were unfed and part fed, respectively. The highest rates of host seeking activity by *Aedes aegypti* occurred soon after dawn and, to a lesser extent, at dusk (Fig 5).

**Fig 6:**
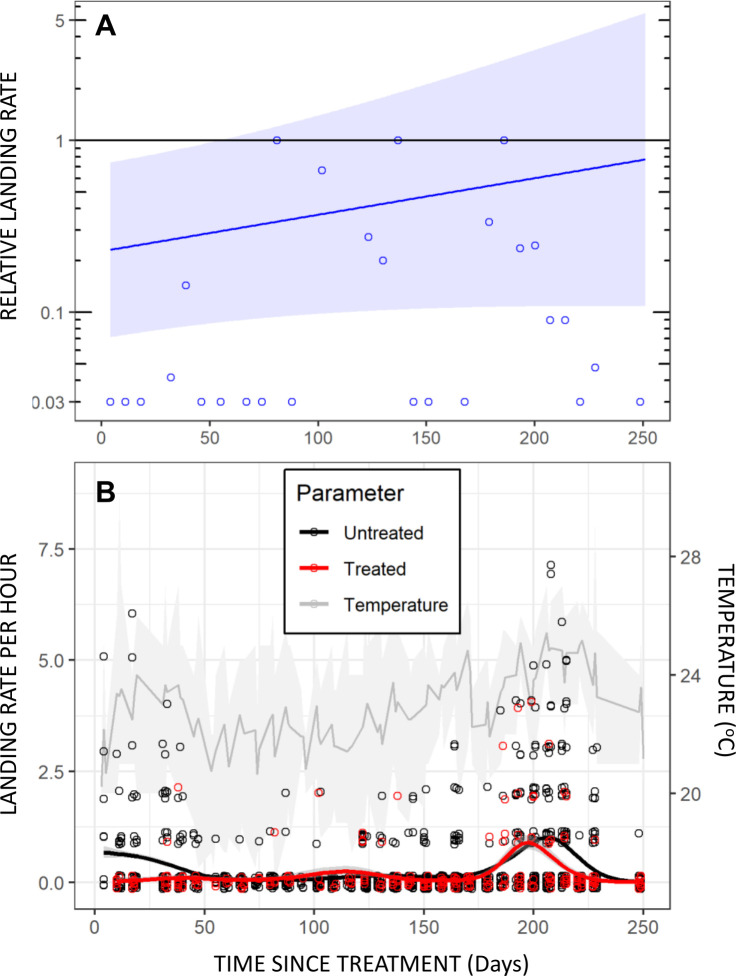
Longitudinal assessment of the protective efficacy of transfluthrin emanators treated with 3g of active ingredient over >8 months, using mosquito electrocuting traps to measure landing rates of *Aedes aeygpti* land [35,36] against wild *Aedes aegypti* populations in open fields in Dar es Salaam. **A**: Effect sizes estimated as relative landing rates on volunteers using treated emanators compared with volunteers using untreated emanators. **B:** Daily summary observations for users of treated and untreated emanators fitted to separate smoothed Poisson-distributed loess regression models and overlaid upon mean, minimum and maximum temperatures for each day of experimentation. The mean trend lines for mosquito catches with treated and untreated emanators were fitted with the non-parametric *locally estimated scatterplot smoothing* algorithm of the *geom_smooth* function in the *ggplot* package of R.

Preliminary analysis of the data from this longitudinal field assessment appear to be broadly encouraging, yielding initial protective efficacy estimates that approached our target of 80% and appeared to taper off slowly over several months (Fig 6A). No evidence for increased landing rates upon nearby non-users that might suggest diversion from users to non-users was apparent (P≥0.23). Indeed, close examination of the raw data in Fig 6B reveals an encouraging picture immediately after treatment, with not a single *Aedes* caught by users of treated emanators over the first month.

However, the confidence intervals around the time trend for estimated protective efficacy were very wide (Fig 6A) because *Aede*s abundance in trap catches were generally low (Fig 6B) in the open fields (this mosquito species strongly prefers shade) required to test for possible diversion over a range of different distances. Furthermore, some landings on users of treated emanators were observed in the second month, following which essentially no mosquitoes were caught for two months (Fig 6B), so the estimated protective efficacy time trend is unsubstantiated over that interim period. Once the *Aedes* population returned to measurable densities, there was no obvious difference between the treated and untreated emanators, and the two smoothed trend lines in Fig 6B largely overlap. The fitted line in Fig 6A may therefore simply reflect a crude average trend from high efficacy at the start to little if any efficacy at the end, with negligible data to inform the model fit in the middle of the time course. Overall, it is concluded that the only clear evidence for satisfactory protection obtained from this experiment is within the first month or so of treatment.

### Comparisons of alternative emanator designs, transfluthrin formulations, mosquito capture methods and emanator deployment positions

**Fig 7:**
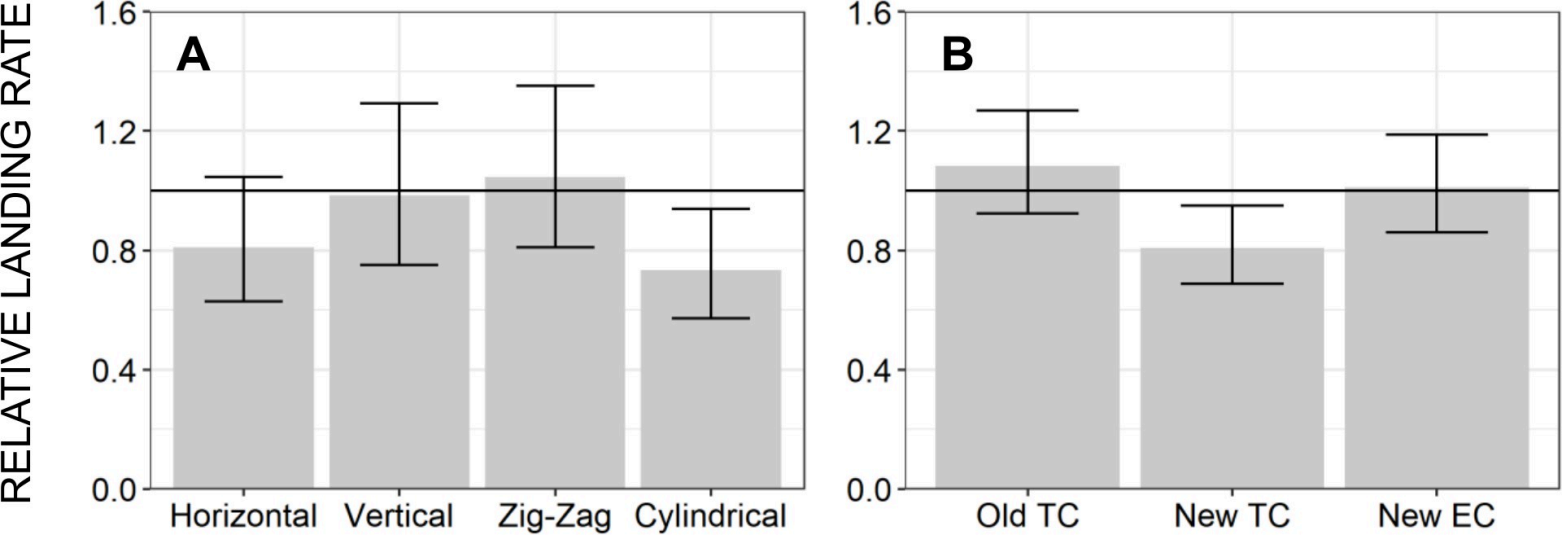
The relative landing rates of wild *Aedes aegypti* upon human volunteers using emanator devices treated with 3g of transfluthrin active ingredient in comparison with users of untreated placebo devices in full field experiments in Dar es Salaam. Landing rates were measured with mosquito electrocuting traps (METs) [35–39] when users were provided with (**A**) four different emanator prototype designs (Fig 3) and (**B**) three different independently shipped batches of formulated transfluthrin for treating the hessian strips (one emulsifiable concentrate (EC) and another two pure technical concentrate (TC) formulations.

**Fig 8:**
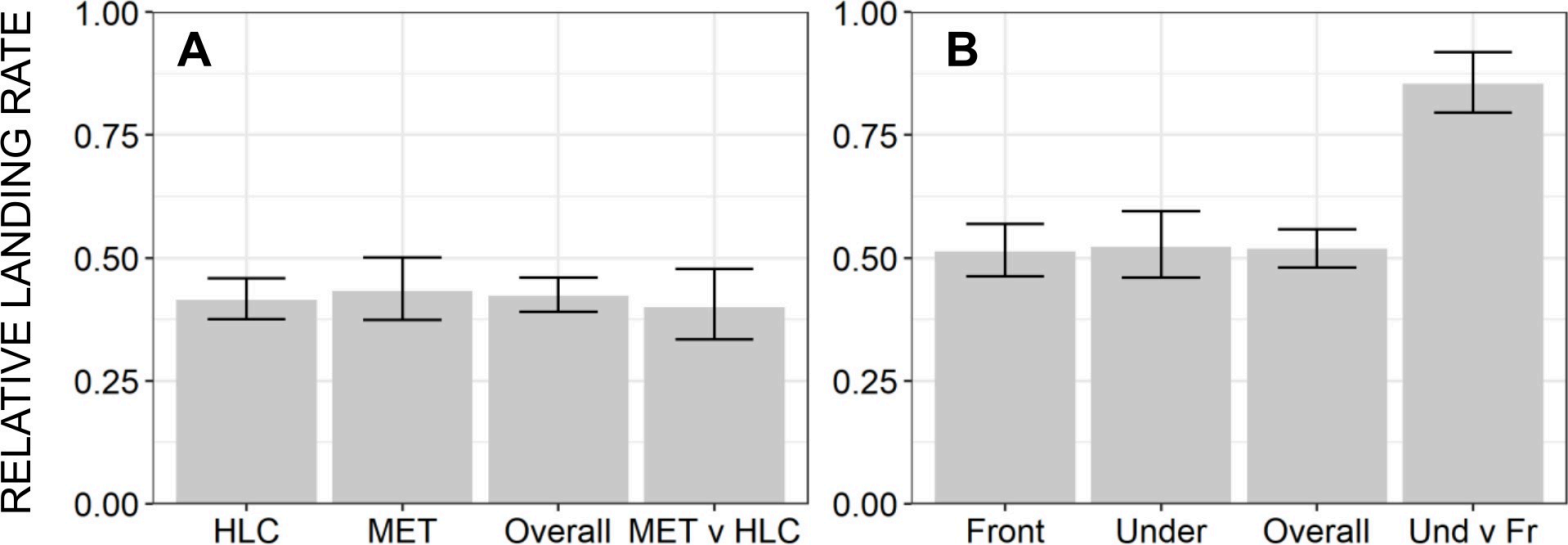
The relative landing rates of insectary reared *Aedes aegypti* upon human volunteers using emanator devices treated with 3g of transfluthrin active ingredient in comparison with users of untreated placebo devices inside a large-cage semi-field system. Landing rates were measured when using a (**A**) two different mosquito capture methods (Mosquito electrocuting traps (METs) [35–39] as per all the other experiments described herein versus the gold standard human landing catch (HLC) method [34], and (**B**) two different deployment positions for the transfluthrin emanators (Placed under the chair of the human user (Fig 2B), as per all the other experiments described herein, or in front of his or her feet.

**Fig 9:**
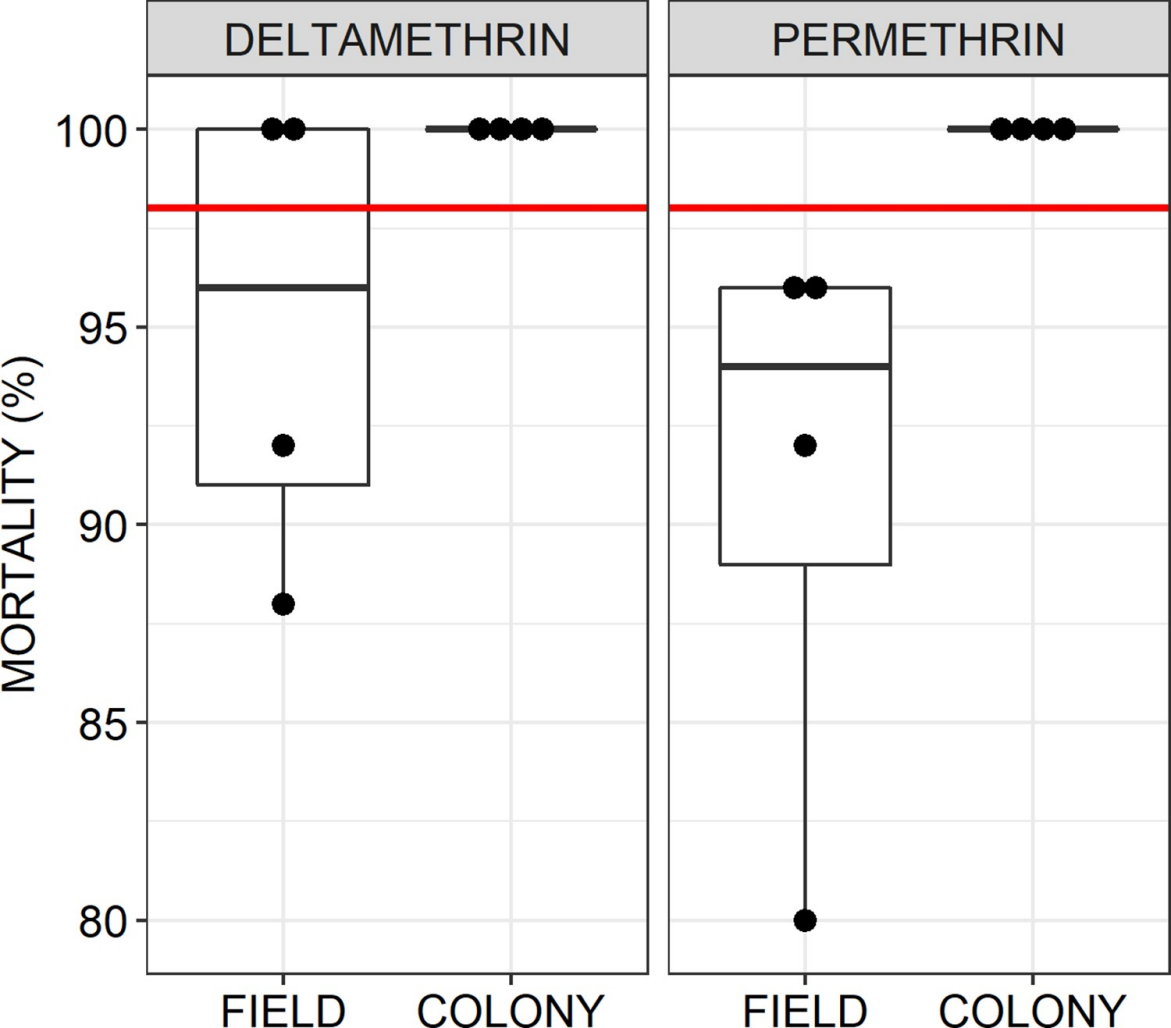
Resistance assay results for wild field populations of *Aedes aegypti* exposed to the pyrethroids deltamethrin and permethrin, compared with a fully susceptible colony of the same species using standard WHO procedures [41]. For the field-sourced mosquitoes, each experimental replicate included at least one distinct independent collection, which was not used in any other replicate, to preclude pseudo-replication arising from genetic relatedness within collected egg batches. The box plot overlain on the individual mean mortality results per replicate assay represent the median and estimated first and third quartiles, while the red line represents the standard WHO minimum threshold considered to represent full susceptibility [41].

Subsequent troubleshooting experiments to assess alternative emanator designs and treatment formulations only served to confirm consistent lack of measurable protection. Satisfactory protection against wild *Ae*. *aegypti* (Fig 7A and 7B, respectively). Protective efficacy as high as 61% was observed in the semi-field large cage experiments using captive reared mosquitoes from an insectary colony (Fig 8A and 8B) which was fully susceptible to pyrethroids (Fig 9), which compares well with the results of others using similar methodologies [34,39,44]. Nevertheless, this fell short of our *a priori* target of 80% protection (Fig 8A and 8B, respectively), and emanators treated with the same 3g dose of transfluthrin consistently achieved negligible reduction of landing rates by wild *Ae*. *aegypti* under full field conditions (Fig 7A and 7B). Overall, neither emanator design (Fig 7A) nor transfluthrin formulation (Fig 7B) nor mosquito capture method (Fig 8A) nor the positioning of the emanator (Fig 8B) were associated with substantially improved emanator performance.

### Comparison of susceptibility to conventional solid phase pyrethroid and responsiveness to transfluthrin vapour at a range of doses in wild mosquito populations and laboratory colonies

Standard WHO bioassays confirmed that the laboratory colony of *Ae*. *aegypti* used was indeed fully susceptible to conventional solid phase pyrethroids, while wild populations in this setting appear to be moderately resistant (Fig 9).

**Fig 10:**
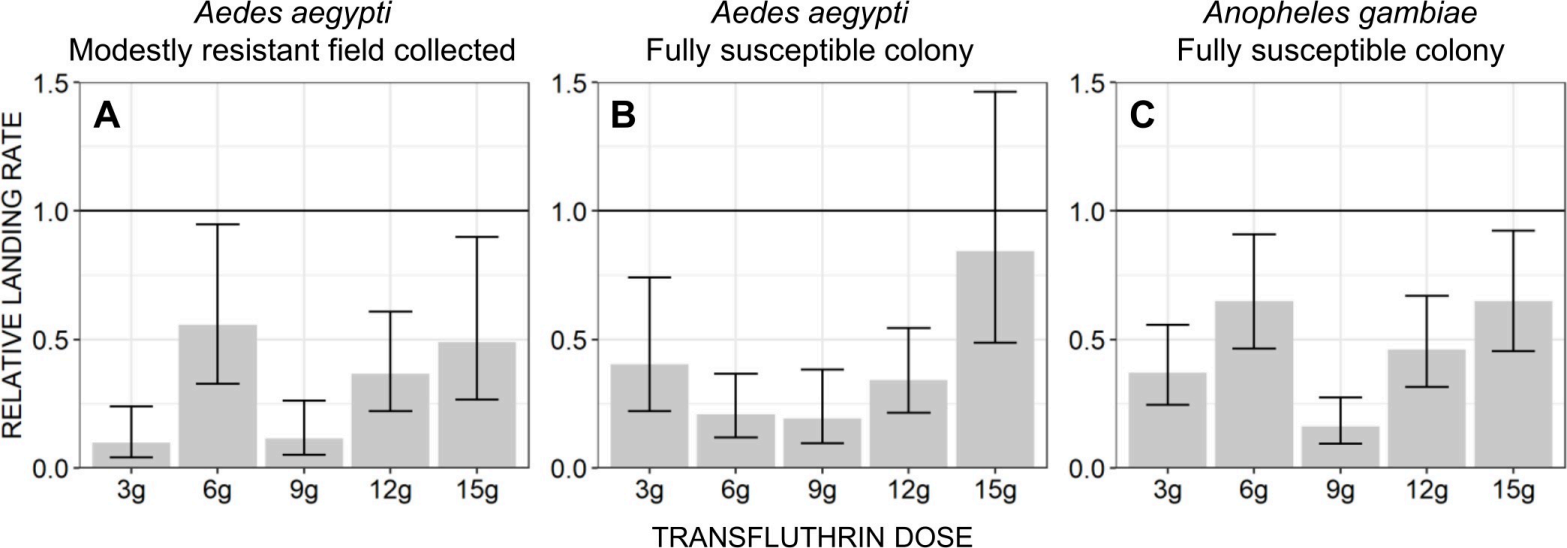
Dose-response relationships for landing rates of mosquitoes on human volunteers using transfluthrin-treated emanators. Mosquitoes from the three following sources were reared alongside each other in the laboratory and then released into a large-cage semi-field system: (**A**) Multiple independent collections (at least one distinct one per experimental replicate, to preclude pseudo-replication) from moderately resistant local field populations of *Aedes aegypti* (Fig 9), (**B**) *Aedes aegypti* sourced from a fully pyrethroid-susceptible laboratory colony (Fig 9), and (**C**) *Anopheles gambiae* sourced from a fully pyrethroid-susceptible laboratory colony. As for all preceding experiments, effect sizes were estimated as relative rates at which *Aedes aeygpti* land on volunteers using treated emanators when compared to volunteers using untreated placebo emanator devices, as measured with mosquito electrocuting traps (METs) [35–39].

While behavioural responsiveness to the transfluthrin emanators was observed for all three mosquito populations assessed in the dose-response experiments (Fig 10), overall the level of protection was similar to those observed in the previous troubleshooting experiments in the same semi-field system (Fig 8A and 8B) and fell somewhat short of our *a priori* target of 80% protection (Fig 10). Across all dosages, transfluthrin emanators exhibited approximately similar protective efficacy against *Ae*. *aegypti* sourced from either moderately resistant stock reared from wild caught eggs (Fig 10A), a pyrethroid susceptible colony of the same species (Fig 10B), and against *An*. *gambiae* sourced from a pyrethroid-susceptible reference colony (Fig 10C). It therefore appears unlikely that the moderate levels of pyrethroid resistance observed in wild *Ae*. *aegypti* populations in this setting can in itself explain the negligible levels of protection exhibited by various emanator formats and transfluthrin formulations under full field conditions (Fig 7). Consistent with Ogoma *et al* 2017 [23], no consistent increase in protection level was observed as the dose increases beyond 3g (Fig 10).

### Transfluthrin vapour concentration in the air around treated emanators

**Fig 11:**
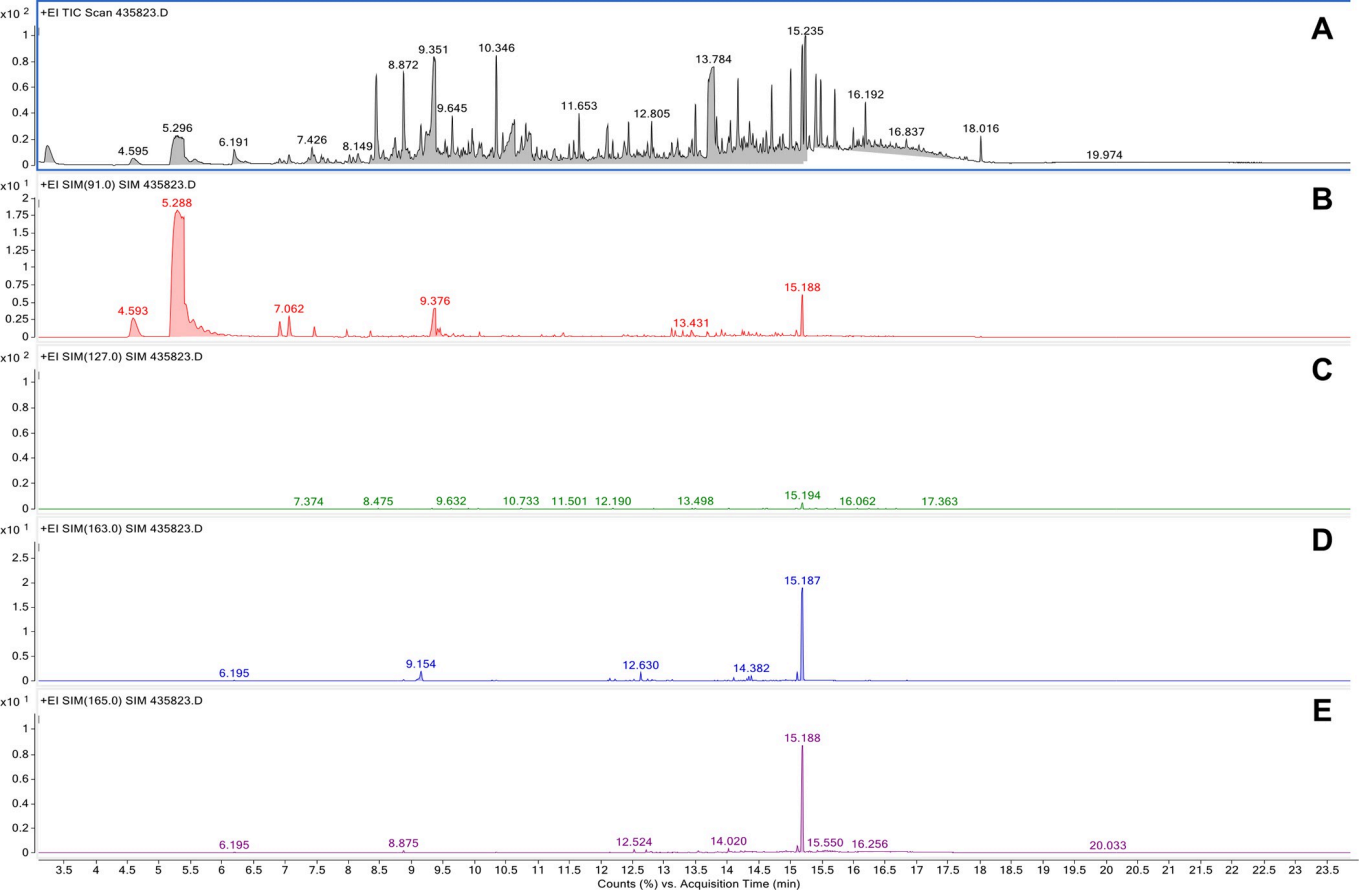
Example chromatograms obtained by GC-MS analysis of an air sample collected in Bagamoyo (second sample detailed in Table 1). (**A**) total ion chromatogram; (**B**) single ion chromatogram for *m/z* 91; (**C**) single ion chromatogram for *m/z* 127; (D) single ion chromatogram for *m/z* 163; (**E**) single ion chromatogram for m/z 165.

The total ion chromatograms obtained from analysis of samples collected in Bagamoyo were more complicated than the laboratory calibration standards. However, as shown in Fig 11, the SIM method is nevertheless capable of singling out transfluthrin, so that both identification and quantification were possible.

The results from the transfluthrin vapour measurement experiments are summarised in Table 1. The average concentration of transfluthrin in the sample tubes ranged from 12.65 to 19.35 μg/m^3^, indicating a relatively consistent emission rate from the emanator. Concentrations were at their highest during the afternoon experiments, suggesting that higher temperatures may indeed increase emission rates of the pyrethroid active ingredient. Overall, these results indicate that transfluthrin vapour concentrations were 25 to 40 times lower than the 500 μg/m^3^ maximum acceptable long-term exposure concentration defined by the regulatory authorities of the European Union [43].

## Discussion

Apart from the encouraging levels of protection observed at the very outset of the initial longitudinal assessment of transfluthrin emanator efficacy (Fig 6A), the results obtained with wild *Ae*. *aegypti* under full field conditions generally fell far short of what was hoped for, with negligible levels of protection observed across all the experiments represented in Figs 6B, 7A and 7B). Some protection was observed against insectary reared mosquitoes, including those derived from wild-caught stock, in large-cage semi-field systems with less airflow than a fully outdoor environment (Figs 8 and 10). Consistent with other reports of similar experiments [34,39,44], estimated protective efficacy within the semi-field experimental system varied between 40 and 90%, but were generally distributed towards the lower end of this range (Figs 8 and 10), thus falling short of the *a priori* target of 80% defined at the outset. The negligible protection levels observed under full field conditions could not be improved upon by changing emanator design (Fig 7A) or transfluthrin formulation (Fig 7B), nor could the modest protection levels observed under semi-field conditions by changing mosquito capture method (Fig 8A) or positioning of the emanator (Fig 8B).

The most obvious possible explanations for the striking contrast between these entomological results and those previously obtained in the same Tanzanian settings by the same investigators [22,23,33] are (1) All the formulations of transfluthrin used herein were different from those used previously and came from different manufacturer, (2) a different emanator prototype design and deployment practice was used here, (3) this is the first time these devices have been evaluated against wild populations of day-biting *Aedes* under full field conditions, and (4) the MET method [35–39] used here for measuring mosquito landing rates under experimental conditions may not accurately reflect human biting rates under normal conditions of routine use. Each of these potential explanations is discussed in detail as follows.

The transfluthrin provided by Bayer/Envu for this study was of such high purity that the technical concentrate formulations was consistently frozen into a white crystalline form at ambient temperatures, whereas the previously-used generic material (Sunrising Company) was brown and was usually at least partially melted into a slushy semi-solid at temperatures well below the melting point of pure transfluthrin. It is therefore possible that the cruder generic material volatilizes more readily for some reason because of the impurity profile, although it should also be noted that the impurities found in such generic sources may also be associated with measurably increased toxicity [45]. Having said that, satisfactory levels of protection against nocturnal *Anopheles* and *Culex* mosquitoes have recently been documented under full field conditions using exactly the same Bayer formulations to treat a variety of alternative prototype hessian emanator formats [46–50], confirming that the source of transfluthrin used here is unlikely to be the main reason for the negligible protection reported herein under full field conditions (Fig 7).

While the mobile rectangular emanator evaluated here differs substantively in shape, size and position from the original prototype, which consisted of a suspended ribbon encircling a seated person [22,23,33], it is notable that several other studies confirm satisfactory efficacy of very similar portable designs to those used herein against field populations of nocturnal *Anopheles* and *Culex* species [51,52]. Alternatively, the related experiences of some community users in Haiti [25] suggests that use of a single emanator device, rather than two, might explain the negligible protective efficacy observed here under similar full field conditions:

*“I had given an emanator* [away]*. I still have one left. When I had two, it was more efficient. Now I only have one. It lacks efficiency.”* Community end user, Haiti [25]

Thus, it seems that the use of two or more emanators might create a more effective protective “bubble” [15,53] in open outdoor spaces, where wind speed and direction can fluctuate considerably over time scales of minutes and even seconds. Having said that, however, recent semi-field assessments of sitting in between two similar self-standing emanators yielded similarly modest estimates of protective efficacy against *Ae*. *aegypti* [34,39,44] to those reported here for a single device. Consequently, the use of one rather than two emanators in the study reported herein seems an unlikely explanation for observed lack of protection against wild, free-flying populations of the same species in the same setting.

Indeed, published evaluations of hessian-based transfluthrin emanators against day-biting *Aedes* have thus far been limited to semi-field systems, yielding similarly modest protective efficacy estimates [34,39,44] to those reported here from the same kind of large cage experiments with captive-reared mosquitoes (Fig 8A and 8B). Notably, similar large cage assessments of a sandal format of transfluthrin emanator indicated protective efficacy against colony-reared *Ae*. *aegypti* was somewhat lower than against *An*. *gambiae* and *An*. *arabiensis* [54,55]. Furthermore, the lack of measurable protection against wild *Ae*. *aegypti* populations under full field conditions in Tanzania reported herein are consistent with those from similar evaluations based on entomological measurements in Haiti [24] and Brazil (Alvaro Eiras, personal communication). In the absence of any evidence to the contrary, face value interpretation of these entomological assessments alone would indicate that these emanator prototypes and transfluthrin formulations appear to provide little if any protection against day biting *Aedes* in these three urban tropical contexts.

More encouragingly, however, complementary social science studies conducted in Port-au-Prince, Haiti contrast starkly with the generally unsatisfactory entomological results from both Haiti and Tanzania, even though these assessments of community end-user satisfaction in Port-au-Prince [25] used exactly the same two batches of the transfluthrin TC formulation from the same manufacturer. These carefully triangulated social science studies used several complementary survey methods, all of which consistently indicated high levels of user satisfaction with the emanators [25]. Similarly, Brazilian end users of a sandal format of transfluthrin emanators developed in Tanzania, which had proven efficacious against *Anopheles* but notably less so against *Ae*. *aegypti* there [54,55], also expressed surprizing levels of satisfaction and narrated their positive impressions in convincing detail, despite disappointing entomological estimates of protection in the same context (Alvaro Eiras, personal communication).

Although the validity of the MET method for collecting human-biting mosquitoes [35–39] has been questioned as a means to quantify the protection provided by vapour phase insecticides like transfluthrin, the traditional gold standard HLC method yielded essentially identical estimates of protective efficacy under semi-field condition (Fig 8A) and similar results have been obtained by others using essentially identical methodologies [34,39,44]. Although the evaluations in Brazil could have been confounded by the use of BG Sentinel Traps^®^ (Biogents AG, Regensburg, Germany) to measure mosquito attack rates, it is notable that this capture method yielded protective efficacy estimates that compared well with HLC and METs under semi-field conditions in Tanzania [39].

However, METs, BG Sentinel Traps and HLC all actually measure the rates at which mosquitoes land rather than bite and feed. It is also notable that transfluthrin and other pyrethroids can incapacitate mosquitoes for extended periods [15,56], that HLCs may underestimate the contribution of this mode of action [34], and that the authors of this article have sometimes observed mosquitoes landing on them but not biting them while they were using similar transfluthrin emanators to those described herein. Thus, although these three capture methods all appear to give consistent efficacy estimates for transfluthrin emanators [39], and the latter also compares well with estimates based on numbers of recovered blood-fed mosquitoes [34], it is nevertheless possible that these approaches may misrepresent the true protective efficacy of these devices. Given the recent epidemiological evidence that another transfluthrin emanator device can protect against *Aedes*-borne arboviruses [57], it may be useful to alternative methodological approaches to measuring actual biting rates under real world conditions, such as field surveys of blood fed mosquitoes [57] or serological indicators of human exposure to mosquito saliva [58–64].

An additional possible explanation may lie in the way that end users deploy transfluthrin emanators under routine conditions of use, as they learned through trial and error what perceived to work best in their home environments. Indeed, some of the Haitian community participants described usage patterns that specifically targeted mosquitoes while resting in sheltered refugia indoors, rather than while actively attacking the end user [25]. Furthermore, similar usage patterns and motivations were shared by some users of a sandal format of emanator [54,55] in urban Brazil; Sometimes when they were not wearing the sandals, users left them in places where they perceived mosquitoes tended to hide (Alvaro Eiras, personal communication). Note, however, that although these Haitian and Brazilian end users appear to have targeted mosquitoes while they were resting in various nooks and crannies inside their houses, the practical end user benefits they emphasized in both settings related to reduced biting rates (Reference 25 and Alvaro Eiras, personal communication). In both Tanzania and Haiti, *Ae*. *aegypti* was only rarely observed indoors, so it is reasonable to speculate that this indoor deployment practice described by some Haitian end users may have been motivated by other mosquito taxa, notably the Southern House Mosquito *Culex quinquefasciatus* that abounds in most urban tropical settings. The last of the three entomological evaluations conducted in Haiti were therefore carried out both indoors and outdoors and mosquito collections were extended well into the hours of darkness [24]. Unfortunately, these efforts to evaluate indoor and outdoor protection against *Culex* in Haiti yielded inconclusive results because of low mosquito densities [24], so it remains to be determined whether this alternative deployment practice may be practically useful.

At this point, however, it is only possible to speculate, as detailed above, whether these transfluthrin emanators may have greater impacts on biting rates than landing rates and/or can reduce densities of resting inside houses. For now, the incongruence of the entomological and social science results obtained across Tanzania, Haiti and Brazil, remains unresolved. Nevertheless, it is encouraging that parallel large-scale assessments of other transfluthrin emanator devices in Iquitos, Peru indicate similar inconsistencies between entomological and epidemiological indicators of effectiveness, with arbovirus incidence rates among humans study participants reduced by 34%, whereas resting densities of blood fed *Aedes* mosquitoes were reduced by only 12% [57]. So, while it remains to be seen whether transfluthrin emanators can be developed into viable products for protecting urban populations against arbovirus transmission by *Aedes* or lymphatic filariasis transmission by *Cx*. *quinquefasciatus*, such unsatisfactory entomological results as those reported herein may not necessarily represent a reliable basis for halting such product development efforts.

As such emanator products continue to be developed and refined, the transfluthrin vapour measurements reported here can help inform that process. The consistency of behavioural dose-response curves across mosquito species and strains observed in these studies in Tanzania (Fig 10), where field populations of *Ae*. *aegypti* were found to be moderately resistant (Fig 9), suggests that physiological resistance is unlikely to have played a major role in those discouraging entomological results, even though these two traits are known to be heritably associated [32]. It also seems that dosage was also not limiting to estimated efficacy across the evaluated range from 3 to 15g. Thus, all the other results with the lowest 3g dose probably remain relevant to potential further development of this technology. This is an important observation because the complementary analytical work to quantify transfluthrin vapour under the worst-case exposure scenario (Immediately downwind from an emanator treated with the maximum 15g dose of transfluthrin, as illustrated in Fig 4 and informed by a photograph shared by a community end user in Haiti [25]), yielded estimates ranging from 12 to 19 μg/m^3^. While this is well below the 500 μg/m^3^ maximum acceptable long-term exposure concentration defined by regulatory authorities in the EU [43], it may be close enough to be a cause for concern. In future work, it may therefore be useful to verify whether devices treated with the lower 3g dose are not only efficacious but also consistently emanate transfluthrin vapour at concentrations of 5 μg/m^3^ or less.

## Conclusions

The striking contrast between these generally discouraging results, together with those from similar entomological assessments against wild *Ae*. *aegypti* in Haiti [24] and Brazil (Alvaro Eiras, Personal communication), and the encouraging perspectives of community end users in these two other settings (Reference 25 and Alvaro Eiras, personal communication), remains to be resolved. Although it remains unclear how effective transfluthrin emanators may be as against outdoor-biting *Aedes*, it is encouraging that rigorous epidemiological evidence for protection against arboviruses in Peru is also somewhat at odds with parallel entomological measurements of end-user exposure in the same context [11]. While the transfluthrin vapour measurements reported here indicate levels of potential inhalation exposure that might be of concern for the 15g dose (Table 1), the entomological dose-response results reported here (Fig 10) indicate that the lower 3g dose was sufficient to achieve the modest levels of protective efficacy that could be demonstrated entomologically in Tanzania. It is also noteworthy that the 3g dose proved sufficient to generate strong end user satisfaction and acceptance in Haiti [25] despite similarly disappointing entomological results there [24]. It may therefore be useful to explore alternative ways of measuring exposure to biting vectors, such as field surveys of blood fed mosquitoes [57] or serological reactivity to mosquito saliva antigens [58–64], and conduct further vapour concentration measurements with greater replication at this lower dose to characterize the benefits and risks of these emanator devices more conclusively. It may also be worthwhile investigating alternative deployment practices by end users in Haiti and Brazil, who appear to have targeted indoor resting mosquitoes like *Cx*. *quinquefactiatus* rather than outdoor biting *Ae*. *aegypti* as envisaged at the outset (Reference 25 and Alvaro Eiras, personal communication).

## Supporting information

S1 Data All the entomological data from field and semi-field assessments of transfluthrin emanator efficacy that were used to generate Figs 5–10. (XLSX)

## Abbreviations

EC: Emulsifiable Concentrate
HLC: Human Landing Catch
MET: Mosquito Electrocuting Trap
TC: Technical Concentrate
